# Poorly differentiated component in gastric pinch biopsies predicts submucosal invasion

**DOI:** 10.1186/1746-1596-9-34

**Published:** 2014-02-20

**Authors:** Sun-Mi Lee, Sun Yang, Mee Joo, Kyoung-Mee Kim, Cheol Keun Park, Soomin Ahn, Byung-Hoon Min, Jun Haeng Lee, Seonwoo Kim, Jong Chul Rhee, Jae J Kim, Gregory Y Lauwers

**Affiliations:** 1 Department of Pathology, The University of Texas Health Science Center at San Antonio, San Antonio, TX, USA; 2 Department of Medicine, Samsung Medical Center, Sungkyunkwan University School of Medicine, #50 Ilwon-dong, Gangnam-gu, Seoul 135-710, Korea; 3 Department of Pathology, Ilsan Paik Hospital, College of Medicine, Inje University, Daewha-Dong, Ilsan-Gu, Goyang-Si, Gyeonggi-Do, Korea; 4 Department of Pathology, Samsung Medical Center, Sungkyunkwan University School of Medicine, Seoul, Korea; 5 Biostatistics Unit, Samsung Medical Center, Sungkyunkwan University School of Medicine, Seoul, Korea; 6 Department of Pathology, Massachusetts General Hospital, Boston, MA, USA

**Keywords:** Gastric cancer, Biopsy, Histologic, Submucosa, Invasion, Endoscopic resection

## Abstract

**Background:**

Endoscopic resection has become standard therapy for selected patients with early gastric carcinoma (EGC). However, the preoperative diagnostic accuracy for excluding submucosal (SM) invasion is not precise. Moreover, histologic features predicting SM invasion in gastric carcinomas (SMiGC) have not been studied extensively.

**Methods:**

Pre-treatment gastric biopsies from 60 patients with SM invasion who underwent endoscopic resection were reviewed and compared to 58 biopsies of lesions confirmed to be intramucosal carcinomas (IMC). For validation of the results, an independent cohort consisting of 616 gastric biopsies confirmed as EGC were analyzed. For statistical analyses, χ-square test, Fisher’s exact test and multiple logistic progression tests were used.

**Results:**

In the biopsy specimens of patients with SMiGCs, differentiated histology, poorly differentiated component, wisps of muscularis mucosa, tumor cribriforming, papillary architecture, desmoplasia and intraglandular eosinophilic necrotic debris (IEND) were observed in 96.7%, 36.7%, 16.7%, 16.7%, 23.3%, 40%, and 46.7% of cases, respectively, while the same features were observed in 100%, 5.2%, 0%, 1.7%, 5.2%, 19%, and 22.4% of biopsies with IMC. In multivariate analyses, poorly differentiated component [odds ratio (OR), 9.59, *p =* 0.002], IEND [OR, 6.23, *p =* 0.012], tumor cribriforming [OR, 4.66, *p =* 0.03] and papillary architecture [OR, 5.52, *p =* 0.018] were significantly associated with the detection of SM invasion. In the validation cohort, poorly differentiated component (*p =* 0.003) and papillary architecture (*p =* 0.008) remained significant.

**Conclusion:**

Poorly differentiated component and papillary architecture are significant histopathologic predictors of SM invasion in pretreatment gastric biopsies of lesions considered for endoscopic therapy. Additional prospective studies are warranted to confirm our findings.

**Virtual slide:**

The virtual slide(s) for this article can be found here: http://www.diagnosticpathology.diagnomx.eu/vs/1588557731103084

## Introduction

Over the last decade, the management options for patients with early gastric cancer (EGC) have increased, largely due to the advent of therapeutic endoscopy, which has been associated with a high cure rate with few treatment-associated morbidities [1]. Endoscopic mucosal resection (EMR) is now established as the treatment of choice for differentiated intramucosal adenocarcinomas (IMC) measuring ≤ 20 mm and devoid of ulceration [2]. Furthermore, the more recently introduced endoscopic submucosal dissection (ESD), which allows dissection along the deep submucosal (SM) layer and facilitates one-piece resection, has led to expanded indications for endoscopic resection. To date, ESD can be offered for IMC regardless of mass size without ulcer, ulcerated IMC < 30 mm in size, or minute SM invasive cancer < 30 mm in size [3]. However, in contrast to most centers in East Asia, ESD has not supplanted EMR in many European and North American centers, where it remains the modality of choice for EGC.

Endoscopic ultrasonography and macroscopic assessment by endoscopy are now widely used not only to diagnose, but also stage EGCs. Although the diagnostic accuracy rate of endoscopic assessment and ultrasonography for SM invasion is reported to be around 72.2% [4]. The number of EGCs with submucosal invasion (SMiGC) unexpectedly diagnosed after evaluation of EMR or ESD specimens has been increasing as a direct consequence of the expansion of indications for EMR and ESD [5]. This is an important development, since SM invasion is the most significant risk factor for lymph node metastasis and thus for the prognosis of these patients. Indeed, a large-scale study has reported that the incidence of lymph node metastasis increases from 2.2% in IMC compared to 17.9% in SMiGCs [6].

To identify pathologic features predicting SM invasion in pre-treatment biopsies, several studies have reported results in esophageal and colonic adenocarcinomas; [7-9] however, no study has been conducted in gastric mucosal biopsies. The aim of this study was to evaluate the histologic characteristics of pre-treatment biopsy specimens to determine whether any histologic feature can accurately predict SM invasion.

## Methods

### Study groups

All the cases were selected from EGCs diagnosed on endoscopic pinch biopsies and treated by EMR between January 2002 and November 2007 at Samsung Medical Center, Seoul, Korea. Patients who presented with synchronous GC were excluded.

The first cohort included 60 patients (45 men and 15 women, age range 46–83 years, mean age 65.2 years) who were diagnosed with SMiGC after EMR. None of these patients had endosonographic evidence of lymph node or distant metastases. For the control group, 58 patients (50 men and 8 women, age range 45–84 years, mean age 63.8 years) confirmed to have IMC by EMR during the same period were included.

The endoscopic gross types of the tumors were classified using the five-tier classification system of the Japanese Research Society for Gastric Cancer [10]. The tumor location was classified as upper third, middle third, or lower third of the stomach.

### Histologic evaluation of biopsies and endoscopic mucosal resections

**Figure 1:**
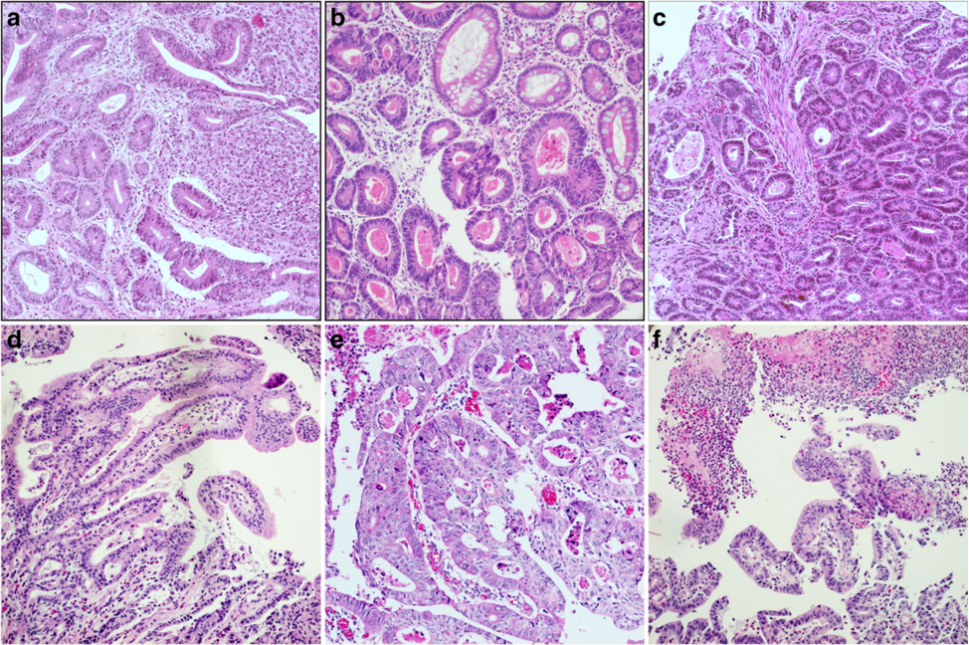
**Representative photomicrographs of pre-treatment biopsies of gastric carcinoma with submucosal invasion.** Sheets of poorly differentiated neoplastic cells within the lamina propria **(a)**; intraluminal amorphous and eosinophilic necrotic cell debris in the lumens of atypical glands **(b)**; small wisps of smooth muscle fibers entrapped within adenocarcinoma **(c)**; papillary structures with a fibrovascular core **(d)**; tumor cribriforming **(e)**; and microscopic ulcer with necrotic exudates **(f)**.

All biopsy specimens were fixed in 10% neutral-buffered formalin, embedded in paraffin, sectioned at 4 μm thickness, and H&E stained. Three to 13 biopsy fragments (mean 5.8 fragments) were available for the SMiGC cases and two to nine fragments (mean 5.1 fragments) for the IMC control cases. For EMR, specimens were pinned to cork mats and fixed in 10% neutral-buffered formalin, and the resection margins were marked by differently colored inks. The fixed specimens were serially sectioned at 2 mm intervals and completely embedded. Three pathologists (Lee SM, Park CK and Kim KM) blinded to the results of the EMRs reviewed all the pinch biopsies and examined them for the presence of specific histologic features described in previous papers on pathologic findings used to distinguish high-grade dysplasia from adenocarcinoma in Barrett’s adenocarcinoma [9,11,12] early colonic carcinoma, [13-15] and gastric carcinoma [16-21]. These features are WHO histologic grade (well, moderately and poorly differentiated); poorly differentiated component (single or small clusters of poorly differentiated carcinoma cells or signet ring cells in 5% to 50% of the tumor volume) (Figure 1a); intraluminal amorphous and eosinophilic necrotic cell debris (IEND) in at least one neoplastic gland (Figure 1b); wisps of muscularis mucosa entrapped within the neoplastic glands (Figure 1c); papillary architecture (Figure 1d); tumor cribriforming (Figure 1e); and microscopic ulcers (Figure 1f). Disagreement in interpretation was resolved by consensus.

Finally, all EMR specimens were reviewed to confirm the diagnoses (IMC versus SMiGC), and the depth of SM invasion was measured as previously described [22]. Briefly, in cases with distorted or lost muscularis mucosa, immunohistochemical staining with desmin was performed, and the lower aspect of the muscularis mucosa was projected by drawing an imaginary line reaching both ends of the discontinuous muscularis mucosa. This line was used as a baseline, and the distance from this line to the deepest point of infiltration was measured as the depth of SM invasion.

### Statistical analysis

Statistical analyses were performed using the Statistical Package for Social Science version 16.0 software for Windows (SPSS Inc., Chicago, IL, USA). For univariate analysis, the χ-square test and Fisher’s exact test were performed, and for multivariable analysis, each predictive pathologic factor of SM invasion was analyzed by multiple logistic regression analyses. Odds ratios and 95% confidence intervals were calculated to estimate the correlation between SM invasion, clinical factors (age, sex, and tumor location), and morphologic findings, e.g., degree of differentiation, microscopic ulcer, poorly differentiated component, wisps of muscularis mucosa, tumor cribriforming, papillary architecture, desmoplastic reaction, and IEND.

### Independent validation cohort

To validate the significant histologic findings in an additional independent cohort, consecutive gastric biopsies diagnosed as adenocarcinoma from January 1 to October 31, 2010 were reviewed by two other pathologists (Joo M and Ahn SM). In the 1497 cases, patients with palliative or multiple cancers, those who were lost to follow-up, or those confirmed as having advanced carcinomas were excluded, leaving a final group of 616 gastric cancers. Among these, the depth of invasion was confined to the mucosa in 423 cases and to the submucosa in 193 cases.

## Results

### Characteristics of gastric carcinoma with submucosal invasion in endoscopic resection

A total of 60 SMiGCs were grossly evaluated by routine endoscopy before EMR. Of these, 34 cases (56.6%) were located in the lower one-third (antrum). The predominant endoscopic findings were combined type IIa and IIc in 28 cases (46.7%), followed by type IIa in seven cases (11.7%) and type IIc in six cases (10%).

### Histologic analyses of gastric biopsy specimens

Table 1 summarizes the incidence of the various pathologic factors present in the biopsies corresponding to cases eventually diagnosed as SMiGC or as IMC. Poorly differentiated component (*p* < 0.0001), entrapped wisps of muscularis mucosa (*p =* 0.0013), cribriforming (*p* = 0.0084), papillary architecture (*p =* 0.0074), desmoplastic reaction (*p =* 0.0157), and IEND (*p* < 0.0001) were significantly associated with SM invasion. However, patient sex, age, tumor location, histologic grade and microscopic ulcers were not statistically associated with SM invasion.

**Table 1:** Frequency of pathologic factors in biopsies from patients with intramucosal carcinomas (IMC) and carcinomas with submucosa invasion (SMiGC)

**Histologic features**	**SMiGCs (%) N = 60**	**IMCs (%) N = 58**	***P value***
WHO histologic grade			
Well	24 (40)	27 (46.5)	0.4472
Moderate	34 (56.7)	31 (53.5)	
Poor	2 (3.3)	0	
Microscopic ulcer	26 (43.3)	17 (29.3)	0.1291
Poorly differentiated tumor	22 (36.7)	3 (5.2)	<0.0001
Islands of muscularis mucosa	10 (16.7)	0	0.0013
Cribriform pattern	10 (16.7)	1 (1.7)	0.0084
Papillary features	14 (23.3)	3 (5.2)	0.0074
Desmoplastic reaction	24 (40)	11 (19)	0.0157
Intraglandular eosinophilic necrotic debris	24 (40)	4 (6.9)	<0.0001

The specificities of poorly differentiated component, cribriforming, papillary architecture, and IEND for predicting SMiGC in EMR specimens were 94.8%, 16.7%, 23.3%, and 93.1%, respectively; and their sensitivities were 36.7%, 16.7%, 23.3%, and 40%, respectively. Combinations of any two or three were associated with a higher specificity but a lower sensitivity (Table 2).

**Table 2:** Specificities and sensitivities of “SM invasion-associated” pathologic factors and combinations of two and three factors

**“SM invasion- related” pathologic factors**	**Specificity (95% CI)**	**Sensitivity (95% CI)**
***Single factors***		
Poorly differentiated tumor	94.3% (80.64 - 98.78)	36.7% (22.04 - 54.25)
Cribriform pattern	98.3% (85.71 - 99.82)	16.7% (7.42 - 33.3)
Papillary feature	94.8% (80.64 - 98.78)	23.3% (11.9 - 40.67)
IEND	93.1% (78.31 - 98.05)	40% (24.76 - 98.78)
***Combinations of two factors***		
Poorly differentiated tumor + IEND	100% (88.58 - 100)	15% (6.38 - 31.38)
Papillary feature + IEND	100% (88.58 - 100)	11.7% (4.42 - 27.31)
Cribriform pattern + IEND	100% (88.58 - 100)	8.3% (2.66 - 23.24)
Cribriform pattern + papillary feature	100% (88.58 - 100)	6.7% (1.88 - 21.06)
Poorly differentiated tumor + papillary feature	100% (88.58 - 100)	5% (1.18 - 18.79)
Poorly differentiated tumor + cribriform pattern	98.3% (85.71 - 99.82)	
***Combinations of three factors***		
Cribriform pattern + papillary feature + poorly differentiated tumor	100% (88.58 - 100)	5% (1.18 - 18.79)
Cribriform pattern + IEND + poorly differentiated tumor	100% (88.58 - 100)	3.3% (0.6 - 11.08)

*Abbreviations*: *IEND* intraglandular eosinophilic necrotic debris.

In multivariate analyses after exclusion of histologic grade, entrapped wisps of muscularis mucosa, and microscopic ulcer, multiple logistic regression analysis corrected by Bonferroni’s method showed that poorly differentiated component [odds ratio (OR) 9.59, *p =* 0.002], IEND [OR 6.23, *p =* 0.0126], cribriforming [OR 4.66, *p =* 0.0318], and papillary architecture [OR 5.52, *p =* 0.0188] were significantly associated with SMiGC.

### Evaluation of the significant histologic findings in the independent validation cohort

In the independent validation cohort, WHO histologic grade (*p* < 0.0001), poorly differentiated component (*p* < 0.0001) and embedded wisps of muscularis mucosa (*p* = 0.0008) were significantly associated with SM invasion. However, cribriforming (*p* = 0.99), papillary architecture (*p* = 0.13), desmoplastic reaction (*p* = 0.09) and IEND (*p* = 0.58) were not associated with SM invasion (Table 3). In multivariate analyses, poorly differentiated component (*p* = 0.003) and papillary architecture (*p* = 0.008) remained significant (Table 4). The diagnostic sensitivity of poorly differentiated component was 48.7% (95% CI, 67.9 ~ 55.8) and specificity was 72.2% (95% CI, 67.9 ~ 76.5). The positive predictive value of poorly differentiated component was 43.5% (95% CI, 36.9 ~ 50.1%) and the negative predictive value was 75.3% (95% CI, 71.1 ~ 79.5).

**Table 3:** “Submucosa invasion-associated” pathologic factors in 616 validation cohorts

**Variables**	**Depth of invasion**		***P *** **value**
	**Submucosa (%)**	**Mucosa (%)**	
WHO histologic grade			<0.0001
Well	114 (59.1)	322 (76.1)	
Moderate	34 (17.6)	47 (11.1)	
Poor	45 (23.3)	54 (12.8)	
Poorly differentiated tumor			<0.0001
Absent	99 (51.3)	301 (71.2)	
Present	94 (48.7)	122 (28.8)	
Islands of muscularis mucosa			0.0008
Absent	160 (82.9)	389 (92.0)	
Present	33 (17.1)	34 (8.0)	
Cribriform pattern			0.9943
Absent	188 (97.4)	412 (97.4)	
Present	5 (2.6)	11 (2.6)	
Papillary architecture			0.1327
Absent	132 (68.4)	314 (74.2)	
Present	61 (31.6)	109 (25.8)	
Intraglandular eosinophilic necrotic debris			0.5814
Absent	128 (66.3)	290 (68.6)	
Present	65 (33.7)	133 (31.4)	
Desmoplastic reaction			0.0934
Absent	190 (98.4)	422 (99.8)	
Present	3 (1.6)	1 (0.2)	
Total numbers	193	423	

**Table 4:** Multiple logistic regression analysis of “SM invasion-related” pathologic factors

**“SM invasion- related” pathologic factors**	**Odds ratio (95% CI)**	***p *** **value**
Poorly differentiated tumor	2.033 (1.271 ~ 3.25)	0.003
Papillary architecture	1.757 (1.158 ~ 2.666)	0.008
Cribriform pattern	1.254 (0.408 ~ 3.857)	0.693
IEND	1.166 (0.789 ~ 1.724)	0.440
Islands of muscularis mucosa	1.489 (0.838 ~ 2.646)	0.175
Desmoplasia	6.054 (0.569 ~ 64.455)	0.136

## Discussion

Endoscopic resection has become standard therapy for selected patients with EGC [23]. Ruling out lymph node metastasis (and the risk thereof) is a critical step prior to attempting EMR or ESD [24]. Lymph node metastasis is rare in small carcinomas with the intestinal phenotype that are confined to the mucosa, while the risk increases with SM invasion [25]. Therefore, endoscopic assessment prior to treatment is crucial for deciding whether the tumor is suitable for EMR [26]. However, the preoperative diagnostic accuracy is not high, and the number of cases with SM invasion detected in the EMR or ESD specimens is increasing [5].

In an attempt to identify histologic features observed in pinch biopsies associated with SM invasion in EMR specimens, we analyzed the cases and found that poorly differentiated component, IEND, cribriforming, and papillary architecture were significantly associated with SM invasion in both univariate and multivariate analyses. However, the degree of differentiation did not always correlate with SM invasion; this is likely a selection bias because most cases that are commonly considered for EMR are the differentiated type. Actually, in the validation cohort, histologic grade was significantly associated with SM invasion. IEND is a distinct pattern of necrosis composed of amorphous eosinophilic material admixed with necrotic epithelial fragments within the lumen of a dilated atypical gland, [27] and it is predominantly found in moderately differentiated gastric adenocarcinomas. In this study, IEND was significantly more frequent in SMiGCs compared to IMCs. Distinct architectural patterns, especially papillary architecture and cribriforming, were also significantly more common in pre-treatment biopsies of cases with SM invasion than in those limited to the mucosa. These two architectural patterns have the same significance in colorectal adenocarcinomas [7,20,21].

In our study, poorly differentiated component was the most significant pathologic factor significantly associated with SM invasion in the validation cohort. This result is consistent with the observations by Kuroda et al., who reported that poorly differentiated component was closely associated with SM invasion in the diffuse type of EGCs and lymph node metastasis [28]. In SMiGCs, poorly differentiated component was reported to be significantly associated with higher risks of lymph node metastasis [19,29]. Zheng et al. also reported that mixed-type gastric carcinomas are larger, more deeply invasive into the wall, and associated with a higher frequency of lymph node metastasis compared to pure intestinal or diffuse-type carcinomas [20].

The present study had several limitations. Pretreatment gastric biopsy may not fully indicate neoplastic progression in a patient with EGC [30]. An endoscopist may miss the invasive component of a tumor that harbors “SM invasion-associated” histologic features. Moreover, although specificity was high, the sensitivity was very low. Furthermore, endoscopic biopsy specimens are small, and thus significant histologic features may be missed. Despite these limitations, multiple biopsies, expertise in endoscopy, and higher resolution endoscopy would be helpful to get higher sensitivity.

In conclusion, poorly differentiated component is the sole significant histopathologic predictor of SM invasion in gastric pre-treatment biopsies and should be evaluated and described in pathology reports.

## Abbreviations

EGC: Early gastric carcinoma; SMiGC: Submucosal invasion in gastric carcinoma; IMC: Intramucosal carcinoma; IEND: Intraglandular eosinophilic necrotic debris.

## Competing interest

The authors have no potential competing interests to disclose.

## Authors’ contributions

SML, KMK and JJK designed the study, collected cases and wrote the manuscript. SML, MJ, SMA, KMK and CKP analysed histological slides. YS, BHM, JHL, JCL and JJK collected the patients’ clinical information, analysed endoscopic findings and obtained the follow-up data. SK performed the statistical analysis. GYL wrote the manuscript. All authors have read and approved the final manuscript.

## Grant Support

This work was supported by a grant from the Samsung Biomedical Research Institute (#SBRISP1B20112).
